# Histological composition and progression of carotid plaque

**DOI:** 10.1186/1477-9560-5-4

**Published:** 2007-02-26

**Authors:** Liz Andréa Villela Baroncini, Antonio Pazin Filho, Simone Gusmão Ramos, Antonio Roberto Martins, Luiz Otavio Murta

**Affiliations:** 1 Department of Internal Medicine, Faculdade de Medicina de Ribeirão Preto, University of São Paulo, São Paulo, Brazil; 2 Department of Pathology, Faculdade de Medicina de Ribeirão Preto, University of São Paulo, São Paulo, Brazil; 3 Department of Pharmacology, Faculdade de Medicina de Ribeirão Preto, University of São Paulo, São Paulo, Brazil; 4 Department of Physics and Math, Faculdade de Filosofia, Ciências e Letras de Ribeirão Preto, University of São Paulo, São Paulo, Brazil

## Abstract

**Background:**

To analyse histological composition and progression of carotid plaque.

**Methods:**

Thirty-one patients (22 males, mean age 68.03 ± 7.3 years) admitted for carotid endarterectomy for extracranial high-grade internal carotid artery stenosis (≥ 70% luminal narrowing) were enrolled. The patients were divided into 2 groups according to symptomatology (group I, 17 symptomatic patients; and group II, 14 asymptomatic patients). A histological analysis and inflammatory cell quantification of each excised carotid plaque was made. Nine carotid arteries were removed from human cadavers that were not preselected for carotid artery disease. These specimens were used as a control tissue without any macroscopic signs of atherosclerotic plaques.

**Results:**

Fifty eight percent of all carotid plaques were classified as complex plaque with possible surface defect, hemorrhage or thrombus. The inflammatory cells concentration did not differ between the two groups. All specimens from human cadavers were classified as preatheroma with extracellular lipid pools.

**Conclusion:**

Asymptomatic and symptomatic patients could have the same histological components on their carotid plaques. Fibrotic and calcific plaques could become vulnerable as complex plaques with surface defect, hemorrhage and thrombus could remain silent. Asymptomatic carotid stenosis should be followed close with no invasive diagnostic methods and clinical evaluation.

## Background

In 1995, a report from the Committee on Vascular Lesions of the Council on Atherosclerosis, American Heart Association (AHA), had described the characteristic components and pathogenic mechanisms of the various advanced atherosclerotic lesions [1]. This report provides a classification of human atherosclerotic lesions based on their histological composition and structure and reflects the temporal natural history of disease. The lesions were classified by Roman numerals that indicate the usual sequence of lesion progression, grading from type I (initial lesions) to type VIII (fibrotic plaque). Varying proportions of different components (connective tissue extracellular matrix; crystalline cholesterol, cholesteryl esters, phospholipids; and cells such as monocyte-derived macrophages, T lymphocytes, and smooth muscle cells) occur in different plaques, thus giving rise to a spectrum of lesions [2,3]. Surface defects, hematoma, and thrombotic deposits futher damage, deform, and thicken, and accelerate conversion from clinically silent to overt disease [1]. The present study was designed to characterize the progression and composition of carotid plaque, according with patient symptomatology, based on the American Heart Association (AHA) classification for human atherosclerotic lesions [1,4]. The inflammatory cell quantification was based on estereology method.

## Methods

### A. Patients

Thirty-six nonconsecutive surgical inpatients admitted for carotid endarterectomy for extracranial high-grade (≥ 70%) internal carotid artery stenosis were entered into this study between February 2003 and July 2005 from 3 participating hospitals. Local ethical committee approval was obtained for the study and procurement of specimens. Written informed consent was obtained from all patients. Exclusion criteria were: a disorder that could seriously complicate surgery (3 patients); terminal cancer (1 patient); and patient refusal of operation (1 patient). The study was conducted on 31 common or internal carotid artery plaques from the 31 remaining patients (22 men and 9 women; mean age 68.03 ± 7.3 years). A clinical examination, including neurological exam, with particular care taken to establish the number and duration of ischemic events, and a record of the time from the last symptom and the operation, was obtained from each patient. Before surgery, all patients underwent a: 1 – either cerebral angiography or magnetic resonance angiography and Duplex ultrasound for grading carotid artery stenosis and assessment of intracranial arterial system; and 2 – either computer tomography (CT) or magnetic resonance brain scan. The presence or absence of infarction in the corresponding middle cerebral artery territory was noted. Focal cerebral ischemic events were defined as transient ischemic attack (TIA), amaurosis fugax (AF), central retinal artery occlusion (CRAO), or cerebrovascular accident. Patients were considered to be symptomatic if they had experienced AF, TIA or stroke ipsilateral to the carotid lesion being studied. Silent infarcts and lacunar symptomatology, diagnosed by a neurologist based on clinical and brain computer tomography (CT) scan and/or magnetic resonance imaging (MRI) located ipsilateral to the stenosis, were also considered symptomatic. On the other hand, patients without any history of recent neurologic symptoms or with nonspecific, nonhemispheric symptoms such as dizziness and vertigo were considered asymptomatic. Each patient was then assigned preoperatively to 1 of 2 groups on the basis of their symptom: group I (n = 17; mean age 66 ± 7 years) symptomatic patients; and group II (n = 14; mean age 67.6 ± 6.81 years) consisting of all asymptomatic patients. At the baseline examination, measurements of height, weight, body mass index, blood pressure, fasting serum total cholesterol, HDL cholesterol, LDL cholesterol, triglycerides, fasting plasma glucose, electrocardiograms and information about coronary artery disease, diabetes mellitus and smoking habits was collected. Percentages of carotid diameter reduction, procedural methods, concomitant therapy, age, sex, and risk factors did not differ between the 2 groups (Table 1). Nine carotid arteries were removed from human adult cadavers that were not preselected for carotid artery disease. These specimens were used as a control tissue without any macroscopic signs of atherosclerotic plaques.

**Table 1:** Patient's Characteristics

	Group I (n = 17) (symptomatic)	Group II (n = 14) (asymptomatic)
Age, years	66.6 ± 6.7	67.6 ± 6.81
Sex, M/F	12/5	9/5
Hypertension	12	13
Diabetes mellitus	6	3
Active Smoking	5	4
Hypercholesterolemia	4	3
CAD	4	4
Aspirin	17	14
Statin	6	5
ACE inhibitors	10	10
Ticlopidine	4	1

CAD = coronary artery disease

### B. Procurement of tissue specimens and histological analysis

Carotid plaques were obtained immediately after endarterectomy. All surgeries were performed with standard surgical techniques, and with minimal manipulation of the specimen. No attempts were made to evaluate the presence and the degree of surface ulceration or thrombus. The plaque should be removed in bloc, without fragmentation or significant distortion. After removal, the section of plaque for histological analysis was placed in fresh 4% paraformaldehyde solution and partly decalcified overnight, in order to be sectioned subsequently. The samples were transected transversely at 3 to 4 mm, and embedded in paraffin. For the most of the specimens, five to six blocks were avaiable. Histological analysis was performed by an experience pathologist (SGR), based on American Heart Association classification for human atherosclerotic lesions [3,4]. The inflammatory cell quantification was assessed by light microscopy with final magnification of 400 ×, using an ocular lens with a grid graticule. The observer selected a region in the plaque section with as many inflammatory cells as possible (hot spot). When no inflammatory area was clearly identified the selection was made in an aleatory way. Total area examined was 0.6 mm^2 ^and each graticule grid corresponds to an area of 0,0625 mm^2 ^with magnification of 400 ×. The inflammatory cells were counted in 10 different graticule area in each specimen. The human carotid arteries removed from cadavers had received the same histological treatment.

### Statistical analysis

Continuous variables were expressed as mean ± SD. Statistical significance was indicated by a value of *P *< 0.05. The comparison of the histological parameters among the groups was done by non-parametric test of Kruskal-Wallis.

## Results

**Figure 1:**
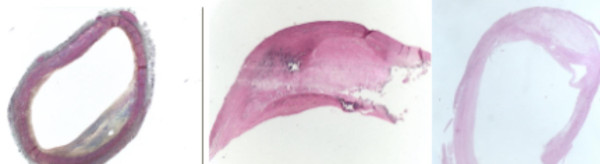
Left panel: Cadaver carotid artery. Type III AHA. Preatheroma with extracellular lipid pools (blue area in the center). Mid panel: Type IV AHA. Atheroma with confluent extracellular lipid core (pink area in the center). Right panel: Type V AHA. Fibroatheroma (clearer pink area – atheroma – surrounded by darker pink area – fibrosis).

**Figure 2:**
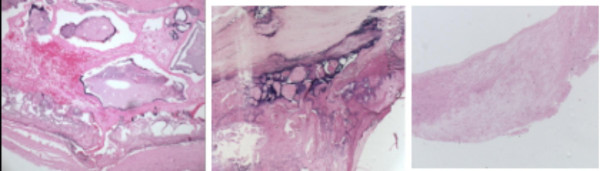
Left panel: Type VI AHA. Complex plaque with possible surface defect, hemorrhage (dotted red area) or thrombus near the lipid core (in the center). Mid panel: Type VII AHA. Calcified plaque (purple areas). Right panel: Type VIII AHA. Fibrotic plaque without lipid core (extensive area of fibrosis).

The histological analysis according to AHA classification for human atherosclerotic lesions is disposed in Table 2. Fifty eight percent of all carotid plaques were classified as Type VI of AHA. All the carotid arteries removed from human cadavers were classified as Type III (preatheroma with extracellular lipid pools). The inflammatory cells counting did not differ between the groups and between the plaque types, varying from 0 to 109 cells by specimen. Figures 1 and 2 are examples of types III, IV, V, VI, VII and VIII of AHA classification.

**Table 2:** American Heart Association Classification for human atherosclerotic lesions according clinical groups.

	Group I (n = 17) (symptomatic)	Group II (n = 14) (asymptomatic)
Type IV	2	0
Type V	4	2
Type VI	7	11
Type VII	2	0
Type VIII	2	1
Inflammatory cells	0–109	0–88
(minimum - maximum/specimen - median ± sd)	(22 ± 28)	(26 ± 29)

## Discussion

An asymptomatic patient with carotid artery stenosis is always a reason of concern for his personal physician. Current AHA guidelines recommended carotid endarterectomy (CEA) for asymptomatic patients, for stenosis 60% to 99%, if the risk of perioperative stroke or death is less than 3%. However, factors in addition to the degree of stenosis, such as the histological composition of the plaque, may be responsible for the determination of stroke risk. The mature atherosclerotic plaque is a complex structure suffering constantly of reparative process and its histological characterization is not easy. It was expected that types IV (atheroma with confluent extracellular lipid core), V (fibroatheroma) and VI (complex plaque with possible surface defect, hemorrhage or thrombus) of AHA classification should be the only ones found in group I (symptomatic patients), but we had classified two patients as having type VII (calcified plaque), and two patients as type VIII (fibrotic plaque). Also, in group II (asymptomatic patients) we found 11 patient as having plaques type VI. The fact that all surgical specimens in the present study had a high grade of complexity shows the challenge to try to separate vulnerable from stable carotid plaques. In the early stages of atherosclerosis the sequence is predictable, characteristics, and uniform, as we could evidence in the carotid arteries removed from human cadavers. However, lesions may subsequently progress in different morphogenetic sequences, resulting in several characteristic lesion types and clinical syndromes [1]. It is well known that vulnerable plaques contain more total lipid and cholesterol, and less collagen and calcium as we could demonstrate in previous study [5-8]. However, the present study had evidenced that the atherosclerotic plaque is not a one-way progression from a "soft" to a "hard" plaque. When or why some these plaques will cross the line between stable to vulnerable? We know that fissures can occur in fibrotic and calcific plaques making then more vulnerable and the present study had evidenced that inflammatory cells are a constant part of atherosclerotic process, even in asymptomatic patients. The answer probably will be in the imunohistochemical analysis [9-14]. Most we have learned about vulnerable plaques is from imaging methods (ultrasound, magnetic resonance, and tomography) that wants to predict vascular events [15-29]. All these methods are able to identify precisely different proportions of fibrous tissue, lipid tissue and calcium as we had demonstrated in previous study [5]. Some methods, mainly ultrasound tissue characterization can classify heterogeneous tissues based on second order statiscal parameters (entropy, homogeneity and energy) but they are failed in determine what this heterogeneous finds really means. Are they related with intrinsic reparative process inside the plaques? All these considerations suggest that asymptomatic carotid stenosis should be followed close with no invasive diagnostic methods and clinical evaluation.

### Study limitations

First, the small number of patients was an important study limitation. This limitation will not be easily overcome, since the improvement of carotid artery stenting techniques will make histological analysis of carotid plaques an infrequent procedure. Second, by necessity, the plaques were sectioned and only a small proportion of each plaque was examined microscopically, and it may well be that features were missed in some patients. Most large lesions vary in composition along their length. This may particularly apply to the classification of fibrotic and calcific plaques in group I, where probably different components were missed when a small number of individual sections were examined. Third, we considered in this study patients with lacunar infarctions (LI) as had symptomatic carotid plaques. According to Tejeda et al [8], although significant carotid stenosis was observed at lower levels in LI, its pathogenic value should be taken into account because, when detected on the symptomatic side, it is not only a marker of atheromatosis but also a process potentially linked to LI. And finally, we did not perform imunohistochemical analysis in the present study that would certainly differentiate more instable carotid plaques, with large macrophage infiltration.

## Conclusion

Mature carotid plaques are complex structures and their histological classification is a real challenge. Asymptomatic and symptomatic patients could have the same histological components on their carotid plaques. Fibrotic and calcific plaques could become vulnerable as complex plaques with surface defect, hemorrhage and thrombus could remain silent. Asymptomatic carotid stenosis should be followed close with no invasive diagnostic methods and clinical evaluation.

## Competing interests

The author(s) declare that they have no competing interests.

## Authors' contributions

LAVB designed the study.

APF also designed the study and made the statistical analysis.

LOMJ also designed the study.

ARM oriented in the histologic analysis of the plaques.

SGR made the histological examination of the carotid plaques.

All authors read and approved the final manuscript.
